# A cost-effective load side management solution based on power line carrier communication i-PLCC

**DOI:** 10.1371/journal.pone.0311313

**Published:** 2024-11-20

**Authors:** Maria Ashraf, Abdul Rafay Khan, Syed Sajjad Haider Zaidi, Asif Gulraiz, Bilal Muhammad Khan

**Affiliations:** Department of Electronic and Power Engineering, National University of Sciences and Technology, Karachi, Pakistan; University of Hull, UNITED KINGDOM OF GREAT BRITAIN AND NORTHERN IRELAND

## Abstract

Conventional power systems are almost at the verge of their existence and are quickly being replaced by modern, especially smarter alternatives. Rapid and wide deployment of Load side management, which is a smarter and modern way to ensure efficient and economical use of power, is a classic example. However, there are several challenges which need to be addressed before it can be realized as a commercially viable solution. Suitable communication method, called “Home Area Networks”, is a mandatory requirement which should be cost effective, robust and with ability to simultaneously handle large number of devices. Due to various inherent issues, communication protocols commonly used now-a-days, are not fully capable to address the requirements of HANs. Power Line Carrier Communications (PLCC) can be a suitable and efficient alternative of quick realization of HANs. In this paper, various aspects of PLCC have been analyzed for use in HANs. Modulation methods, materials, physical parameters and effect of noise have been analyzed. The paper evaluates performance and effectiveness of FSK modulation with aluminum transmission medium for PLC within a residential environment, focusing on the tradeoffs between distance, transmission medium diameter, and type of transmission medium, and cable gauge, which can degrade the performance of PLCC. A novel simulation model has been presented incorporating RLCG (Resistance, Inductance, Capacitance, and Conductance) tools and additive white Gaussian noise (AWGN). Performance parameter have been comprehensively analyzed of implementation using aluminum as a communication medium. This paper discusses cost effectiveness of PLCC by replacing copper by aluminum as the transmission medium. The study reveals that aluminum cables must be twice the size of copper cable in order to achieve same communication distances. Higher carrier frequencies in FSK modulation increase noise susceptibility, consequently reducing achievable communication distances. Cables with smaller diameters results in lower transmission ranges. These results highlight the trade-offs involved in implementing FSK for in-home PLCC (iPLCC). The study highlights how crucial it is to take these trade-offs into account while developing and refining FSK-based iPLCC systems for use in smart homes.

## I. Introduction

**Fig 1:**
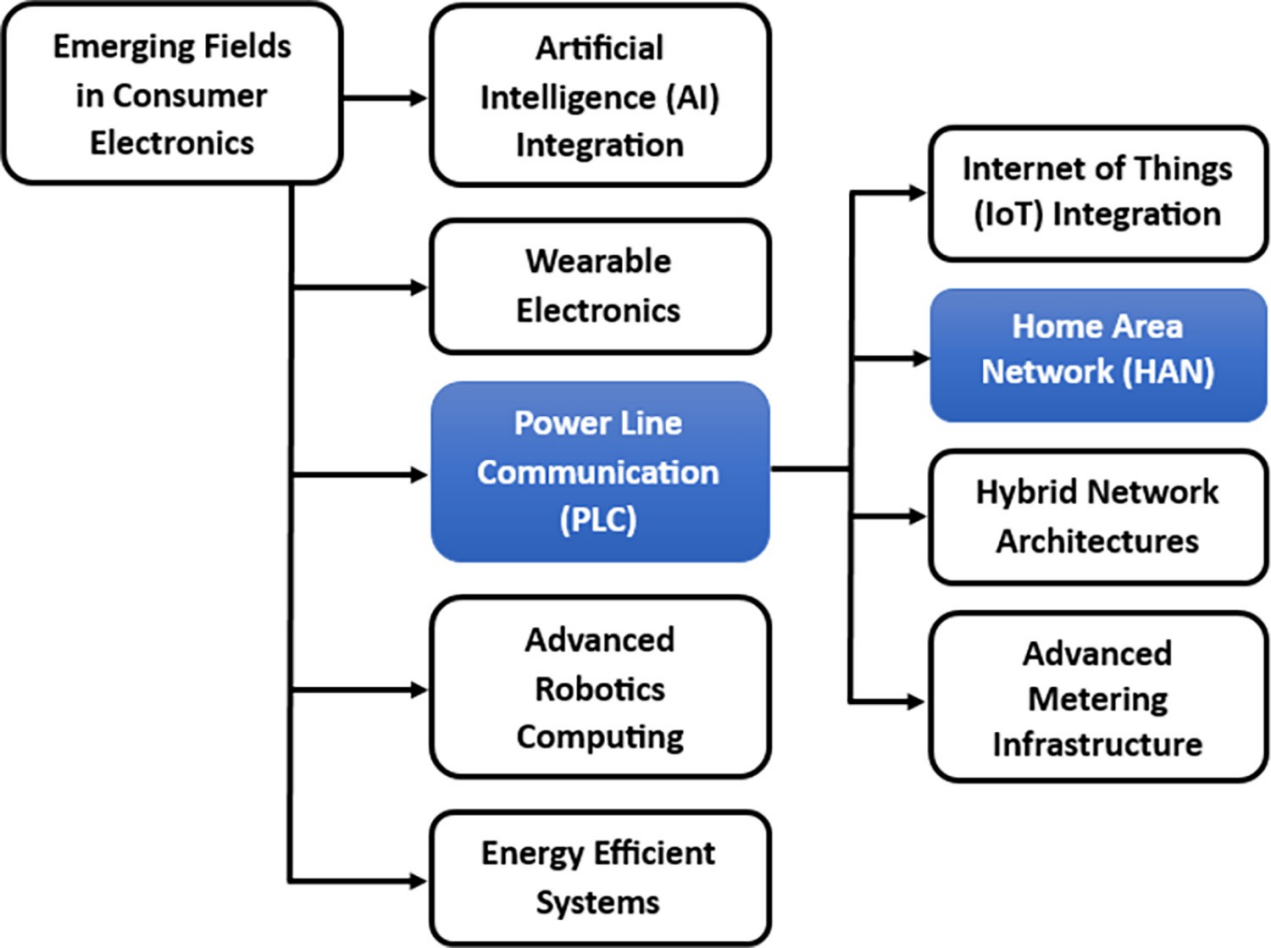
Consumer electronic applications.

In a period defined by the advancement of intelligent domiciles and the Internet of Things (IoT), the Home Area Network (HAN) has surfaced as a crucial component of our everyday routines. Within this context, Power Line Carrier (PLC) communication technology offers an attractive strategy for seamless connectivity, integrating Consumer Electronics (CE) with pre-existing electrical wiring to facilitate data transmission at a lower cost. Fig 1 delineates certain CE applications; nevertheless, a thorough evaluation of PLC is imperative prior to its integration into the HAN system. PLC technology facilitates the smooth assimilation of sensor data exchange, cameras, and various consumer gadgets by making use of the established electricity grid infrastructure [1]. It capitalizes on the existing power line infrastructures that are originally designed for the conveyance of AC electric power in order to enable data transfer [2].

This novel methodology enhances data transmission efficiency through the utilization of current electrical infrastructures, providing opportunities for integration in smart metering, live energy tracking, and remote device management [3]. These contributions support the development of a more interconnected, autonomous and environmentally-friendly future [4,5].

Power Line Carrier (PLC) communication demonstrates significant promise for potential future utilization across a broad spectrum of industries. These industries encompass support for vehicle-to-grid (V2G) systems, the establishment of a resilient home area network, facilitation of electric vehicle charging infrastructure, optimization of smart grid operations [6],smart grid integration with wind energy [7], advancement in medical equipment technology, enhancement of agricultural practices, and provision of Broadband internet access through power lines [8].

The realm of Power Line Communication (PLC) has demonstrated significant advancements in addressing challenges related to noise, attenuation, and channel impairments. Various research endeavors have put forth enhanced models characterized by superior designs, increased data transfer speeds, and cost-efficient resolutions. The current emphasis on broadband PLC has resulted in practical field experiments, exemplified by a study scrutinized in [9], which have spurred the development of innovative transmission methodologies. A recent approach in PLC systems in Greece involves enhancing the effectiveness and dependability of the power grid by integrating and overseeing dispersed energy resources. The integration of smart meters has progressed notably through G3-PLC networks [10], enabling seamless interaction with energy meters through UART technology. Furthermore, the deployment of Advanced Metering Infrastructure (AMI) systems has experienced enhancements, including strategic placement of data collectors in accordance with the PRIME standard [11]. Tables 1 and 2 offer a comprehensive analysis of recent PLC research, shedding light on specific elements crucial for the assessment of PLC performance within a household network.

**Table 1:** Table of related works.

Title	Research	Methodology	Results
Impact of Channel Disturbances on Current Narrowband Power Line Communications and Lessons to Be Learnt for the Future Technologies [12]	in this paper a model for testing the PLC technology using the clone of possible channel disturbances. These clones are somehow are very accurate according to real time channel disturbances that might occur in Powerline communication.	This paper develops a replicable, fully automated, and cost- optimized test scenario, based on an innovative Virtual PLC Labora tory	The results obtained provide important conclusions about the impact of different types of noise on narrowband PLC
A Frequency Control Strategy Using Power Line Communication in a Smart Micro-grid [13]	Presented Power line communication for frequency control in a 15 bus smart micro-grid by changing frequency for transmitting information along with reference signal from the control center to the diesel generator.	The information transmitted at 500 kHz by Mary ASK is decoded on the receiver end for frequency regulation. Physical layer analysis is achieved for Mary FSK and Mary ASK by investigating the average achievable rate and symbol error rate.	It is concluded that Mary ASK is a better choice for signaling in PLC for frequency control.
In-home power line communication channel: Statistical character- ization [14]	compute the impulse response of channel, RMS delay spread, the deterministic coherence bandwidth	Studied wide set of measured channels in the 1.8–100 MHz frequency band	The achievable rate decreases with distance
Measurement Methods of Outdoor Low-Voltage Cable Characteristics for Narrowband Power Line Communication [15]	A low voltage cable characterization for power-line communication in the frequency band of 9 KHz to 500 KHz is presented	The cable losses are investigated.	The characteristic impedance of underground cable is more, and losses in underground cable increase with frequency.

**Table 2:** Comparisons of PLC based application.

Authors	Modulation Type	Frequency Range	Data Rate	PLC Standard	Distance Covered	Distribution Type
L.R.M. Castor [16]	OFDM	2–34 MHz	200 Mbps	Undefined	180 m	Low Voltage
P.J. Pin˜ero [17]	OFDM	2–28 MHz	10–75 Mbps	Home Plug AV	Undefined	Low Voltage
A.G. Merkulov [18]	OFDM	2–30 MHz	24 Mbps	Home Plug AV	700 m	Low Voltage
M.Orgon [19]	OFDM	tens of MHz	622–766 Mbps	Home Plug AV2	300 m	Low Voltage
W.R.S. Osman [8]	OFDM	2–32 MHz	95.14 Mbps	Home Plug AV	150–200 m	Low Voltage
G. Hallak [20]	OFDM	2–30 MHz	95.5 Mbps	ITU-T G.hn	300 m	Low Voltage
P. Mlynek [21]	OFDM,ROBO, DBPSK DQPSK,D8PSK	3–500 kHz	6.4–47 kbps	G3-PLC	1000 m	Low Voltage
N.Uribe-Pe´rez [22]	OFDM	*<*500 kHz	4.5 kbps	PRIME	Undefined	Undefined
Si Chen [23]	QPSK	Upto 120 kHz	1 kbps	RS 485	350 m	Low Voltage
A. Cataliotti [9]	FSK	50–150 kHz	4.8 kbps	Undefined	250–2500 m	MV to LV
S. C. Hsieh [24]	OFDM	140–470 kHz	47.5 kbps	IEEE	500 m	LV to MV
S. Buayairaksa [10]	OFDM	9–490 kHz	100–380 Kbps	G3-PLC	500–1500 m	Low Voltage
P. Mlynek [25]	FSK	36–90 kHz	19.2 kbps	G3-PLC	1000 m	Low Voltage
F. Aalamifar [11]	Adaptive	42–471 kHz	79–388 kbps	PRIME	Undefined	Low Voltage

Power line communication (PLC) is being recognized as an innovative alternative for data transfer in consumer electronics (CE) devices. Every device exhibits the ability to transmit data even in the presence of high voltage. Nevertheless, the standardization of PLC communication technologies may exhibit variations across different countries with regards to limitations on frequency bands.

This study assesses the performance of a PLC system through the development of a precise PLC model designed for the typical Narrowband PLC bandwidth ranging from 3 to 148.5 KHz. An indispensable performance evaluation aids in the enhancement of cost-effectiveness, coverage, and signal integrity. Central to this investigation is the utilization of a Simulink model to appraise the performance of copper and aluminum cables in Power Line Carrier (PLC) communication employing Frequency Shift Keying (FSK). The comprehensive analysis carried out by this Simulink model on diverse cable materials renders it highly relevant and beneficial for the optimization of PLC communication systems. Furthermore, the insights provided by the analysis are anticipated to be advantageous for both smart homes and industries, offering a holistic perspective on cutting-edge technology, outlining challenges and constraints, and proposing avenues for enhancement through experimentation with various cable configurations.

Prior studies on Power Line Communication (PLC) systems in Home Area Networks (HANs) predominantly relied on analytical models with a focus on copper wiring. The objective of this study is to develop a model for Power Line Communication over Copper (PLCC) system that incorporates realistic transmission channel characteristics found in home networks, which can be valuable for practical implementations. Additionally, this research delves into assessing the efficacy of PLC technology within a Home Area Network by investigating the impact of various factors such as line length, carrier frequency, wire material, and other physical parameters.

The structure of this manuscript consists of several sections. Section I delineates the introduction of the Power Line Communication (PLC) system along with various international standards pertaining to power line carrier communication. Section II provides an overview of the literature regarding PLC technology, including a comparative analysis of works by different scholars and researchers. The methodology is expounded upon in Section III. Subsequently, Section IV showcases PLC channel modeling using Simulink. Section V elaborates on the results derived from simulations on the efficacy of PLC technology. Finally, Section VI culminates the performance analysis of PLC technology.

## II. Power line communication system

**Fig 2:**
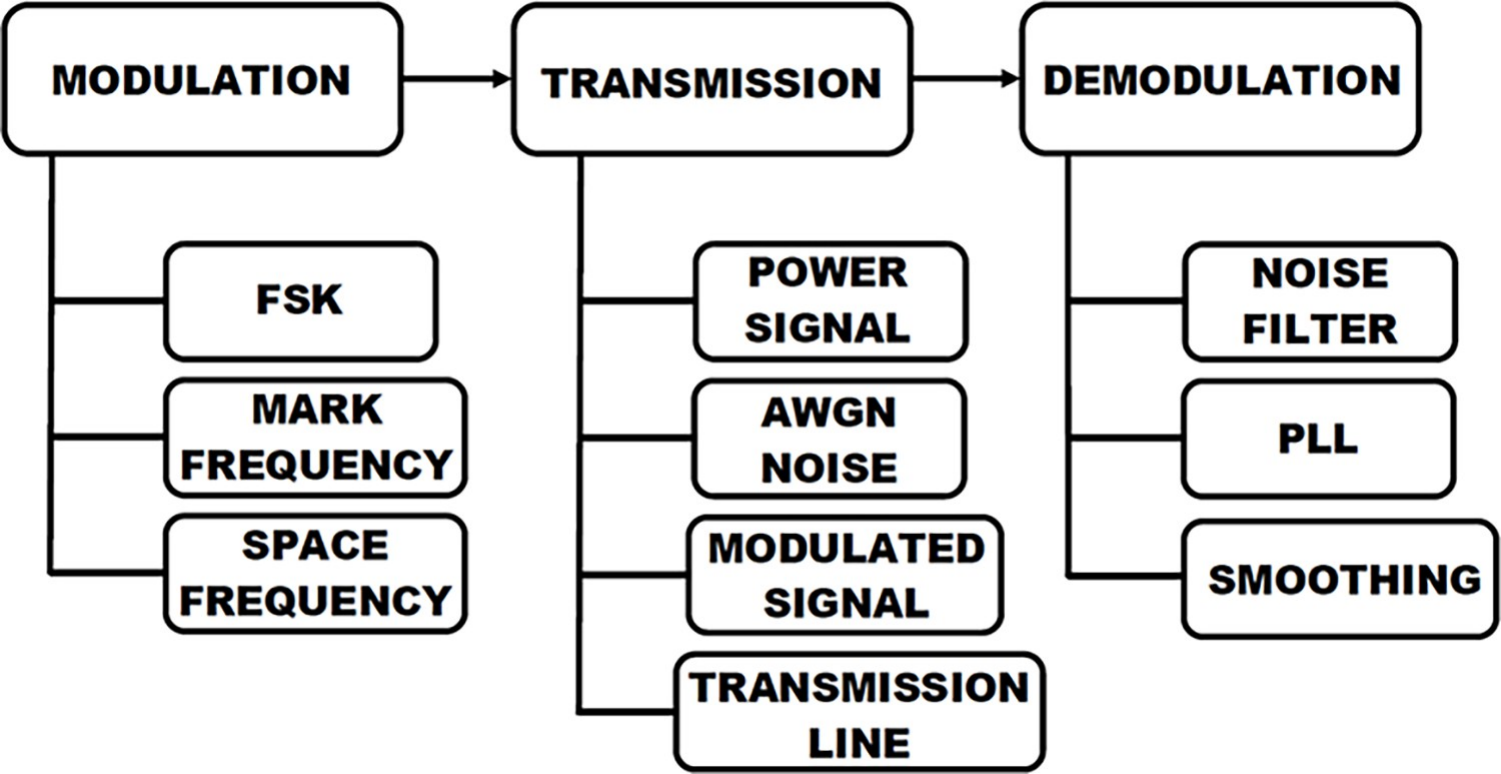
Block diagram of PLC technology.

The power system being analyzed is a single-phase system operating at 220 V and 50 Hz as illustrated in Fig 2. The diagram depicts the comprehensive configuration of the proposed Power Line Communication (PLC) system, comprising three fundamental stages of the communication system. This system has been adapted and formulated in accordance with PLC technology in Simulink MATLAB. This document introduces a proficient approach for transmitting two distinct frequency signals through Frequency Shift Keying (FSK) modulation on a common power line. A Simulink model is devised in MATLAB incorporating an FSK modulator, a transmission line, AWGN noise, and a Phase-Locked Loop (PLL) demodulator. The higher frequency signal, known as the "carrier signal," is analog and conveys the digital data embedded within it. Furthermore, alongside the carrier signal, the 50 Hz AC signal is conveyed on the same line utilizing FSK modulation, which is considerably lower in magnitude than the carrier signal. In practical scenarios, PLC channels are susceptible to noise; hence, AWGN noise is introduced into the transmission channel to emulate the real-world implementation of PLC technology in residential settings. Fig 2 illustrates the elimination of noise from the carrier signal prior to transmission to the PLC demodulator.

## III. Modeling and simulation of powerline carrier communication technology

**Fig 3:**
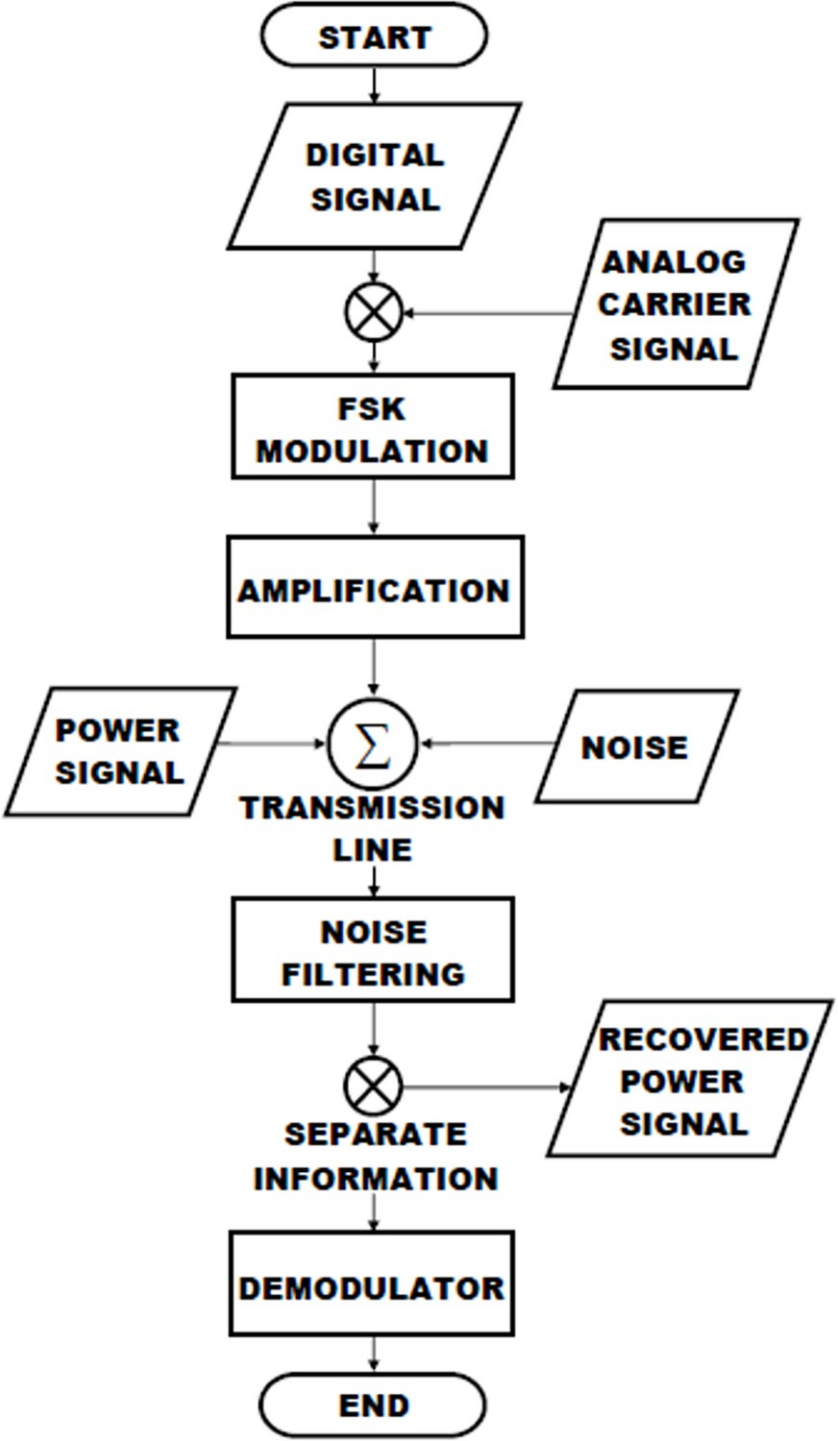
Flowchart of PLC technology.

The suggested system is comprised of the tangible framework of the PLC channel model. Power line communication channels are composed of three primary elements, specifically the Transmitter, Transmission channel, and Receiver. The Simulink model depicted in Fig 3 illustrates PLCC communication utilizing FSK modulation and demodulation, in association with a power signal conveyed to a transmission channel possessing characteristics akin to those of a real transmission line with RLCG parameters.

### A. Modulation

**Fig 4:**
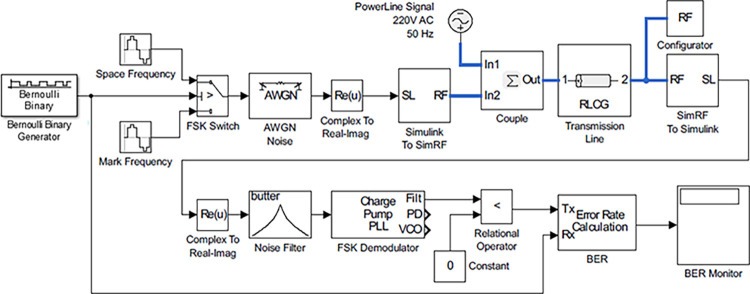
Power line carrier communication model.

In this paper, FSK modulation is used as ASK modulation mostly gets attenuated by noise [26,27]. Also, it is simpler and requires less expensive hardware [28]. In Fig 4 the carrier signal generated from the reference of the information signal is shown. Frequency Shift Keying is expressed by the following equation.


$$V_{fmod}(t)=V_{c}\;cos\;2\pi [f_{c}+V_{m}(t)\Delta f]t$$ (1)

In this paper the mark and space-frequency for the Simulink model are obtained by

$$f_{space}=0.9f_{carrier}$$ (2)

$$f_{mark}=1.1f_{carrier}$$ (3)

### B. In-home transmission channel

**Fig 5:**
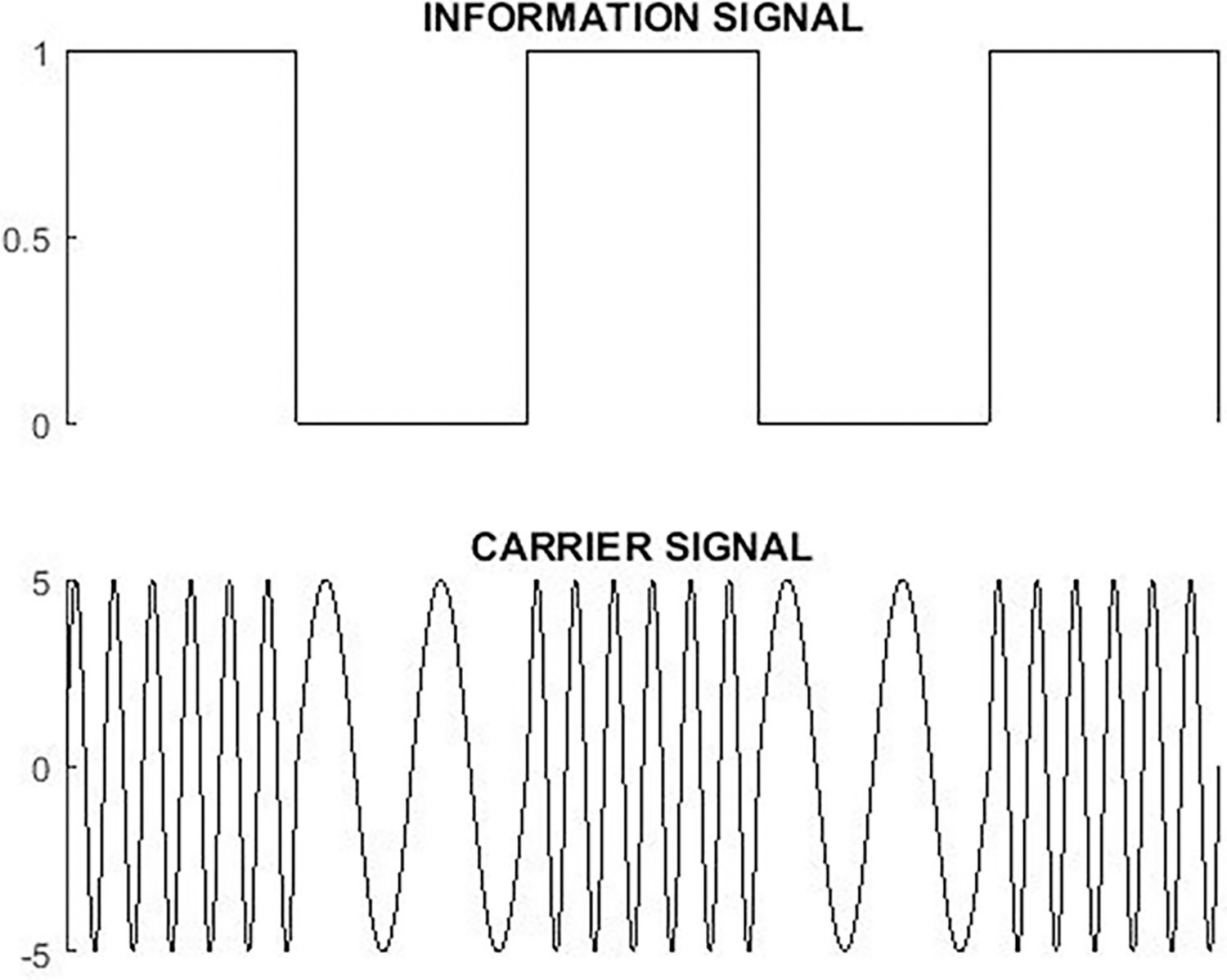
Frequency shift keying modulation.

In-home environments, transmission lines efficiently deliver electric power to devices, making PLC a versatile choice for device interconnection [29,30]. Performance analysis of PLC is conducted using an electrical design model in MATLAB/SIMULINK as shown in Fig 5.

Key equations considered are:

1. Resistance ‘R’ of the transmission line:

$$R=\rho \frac{l}{a}$$ (4)

2. Two-wire transmission line loop inductance:

$$L=4\times 10^{-7}ln\frac{d}{r}\;\frac{H}{m}$$ (5)

3. Potential difference w.r.t neutral:

$$V_{ab}=\frac{q}{\pi \varepsilon }ln\frac{d}{r}volts$$ (6)

4. Capacitance to neutral for the two-wire line:

$$C_{n}=\frac{2\pi \varepsilon }{ln}\frac{D}{r}\frac{F}{m}$$ (7)

5. Conductance ‘G’ (usually negligible):

$$G=\frac{2\pi \varepsilon \prime \prime }{ln}\frac{D}{r}\frac{F}{m}$$ (8)

Parameters for copper and aluminum cables are determined from tables in [31], considering conductor size, strands, area, outside diameter, diameter, and geometric mean radius (GMR) from Tables 3 and 4. Copper’s resistivity is 10.37 Ω-cmil/ft, and Aluminum Conductor Steel Reinforced (ACSR) is 17.00 Ω-cmil/ft.

**Table 3:** Copper cable properties.

Size (AWG)	Strands	Area (Cmills)	Diameter (inches)	Outer Dia (inches)	GMR (ft)
4	3	41740	0.1180	0.254	0.00717
6	3	26250	0.0935	0.201	0.00568
8	1	16510	-	0.1285	0.00417

**Table 4:** Aluminium (ACSR) cable properties.

Size (AWG)	Strands	Area (Cmills)	Diameter (inches)	Outter Dia (inches)	GMR (ft)
2	6+1	77345	0.1052	0.198	0.0039
4	6+1	48765	0.0834	0.250	0.0026
6	6+1	30630	0.0661	0.316	0.0039

The Resistance, Inductance, Capacitance, and Conductance are calculated for the wire configurations for different diameters shown in Tables 5 and 6. The per meter values of RLCG are given in the table and should be multiplied as per the actual length of wire required. Throughout, the two different types of wire will used to analyze the performance of PLCC in a home.

**Table 5:** Transmission line parameters (copper).

Size (AWG)	*R* (Ω)	*L* (*μ*H)	*C* (*p*F)	*G* (*μ*S)
2	0.00072	2.86	9.358	105.69
4	0.00116	3.02	9.008	101.74
6	0.00185	3.19	8.68	98.06

**Table 6:** Transmission line parameters (ACSR).

Frequency	Length (meters) Copper Aluminum (ACSR)					
	4AWG	6AWG	8AWG	2AWG	4AWG	6AWG
3	870	750	580	900	740	610
9	830	630	520	850	600	560
20	800	510	430	820	550	500
60	650	400	390	700	410	395
95	610	370	300	635	380	330
125	450	320	270	480	325	280
135	420	300	240	430	315	255
148.5	380	290	210	385	300	215

Locked Loop (PLL). For the PLL loop filter, a Butterworth low pass filter is used.

**Fig 6:**
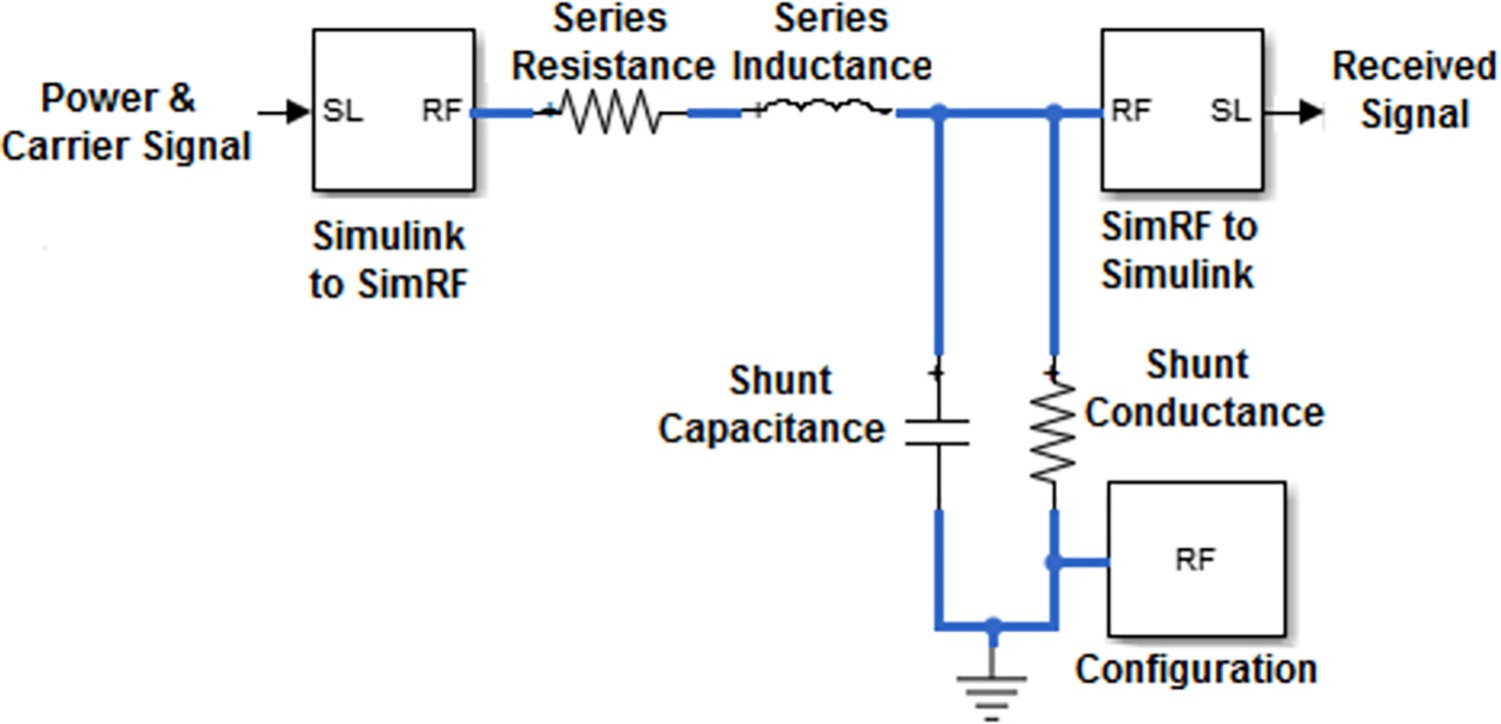
RLCG SimRF channel model.

**Fig 7:**
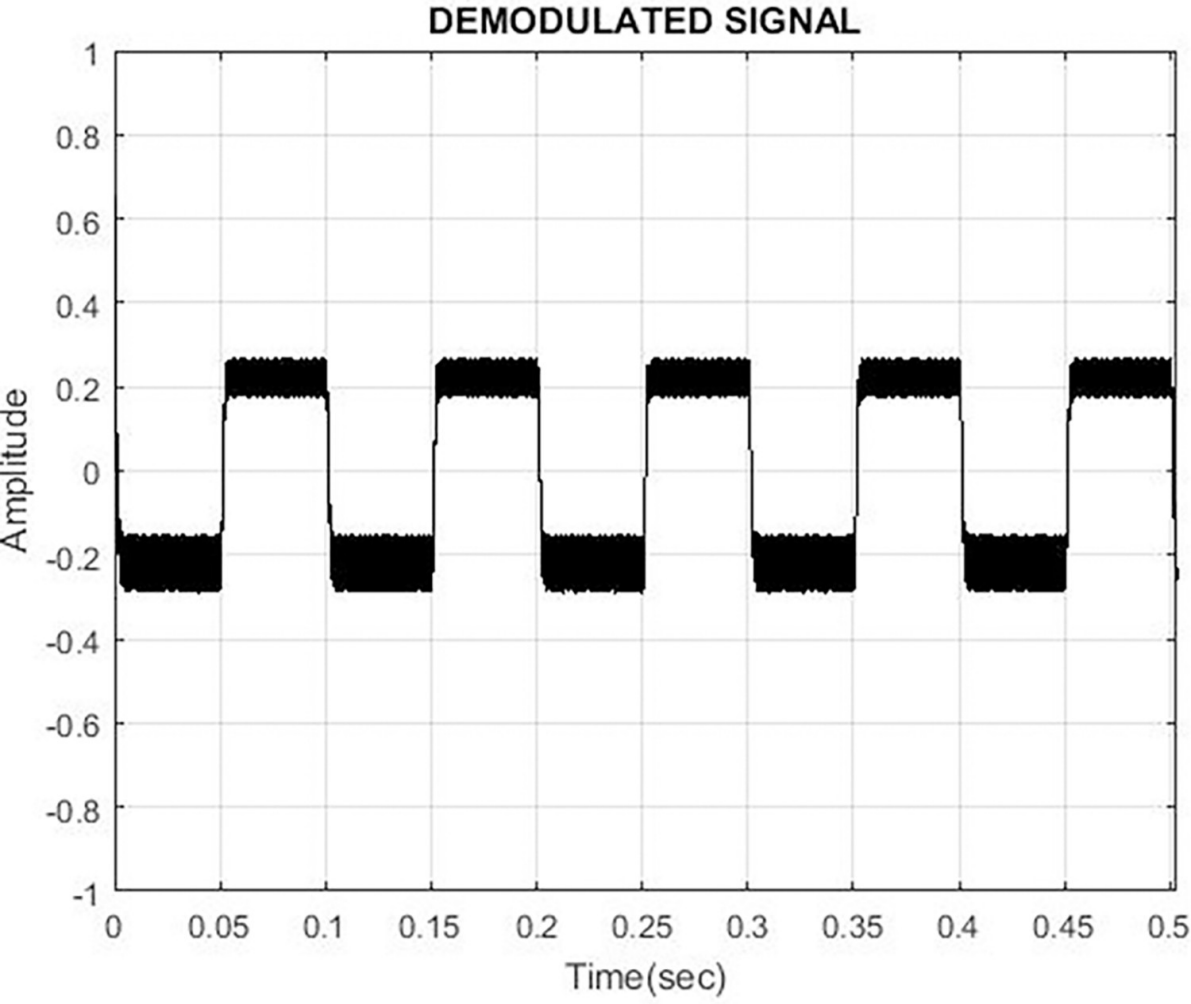
PLL demodulation output.

The output from the loop filter of the PLL FSK demodulator is shown in Fig 6. Increasing the order of the filter increases the settling time, overshoot, and cost as shown in Fig 7. Therefore order of filter for the PLC model is kept 2 having cutoff frequency in angular form.

### C. Cost analysis of aluminium and copper

The study has been carried out that suggests based on real data that over the period from 2011 to 2023 there is an increasing trend in price of copper and Aluminium, however despite this trend Aluminium price in USD per 1000 kg in 2023 is around 2000 USD whereas copper in comparison is at 8000 USD. Therefore, Aluminium beats copper in terms of price and proves to be cost effective as claimed in [32].

## IV. Results

**Fig 8:**
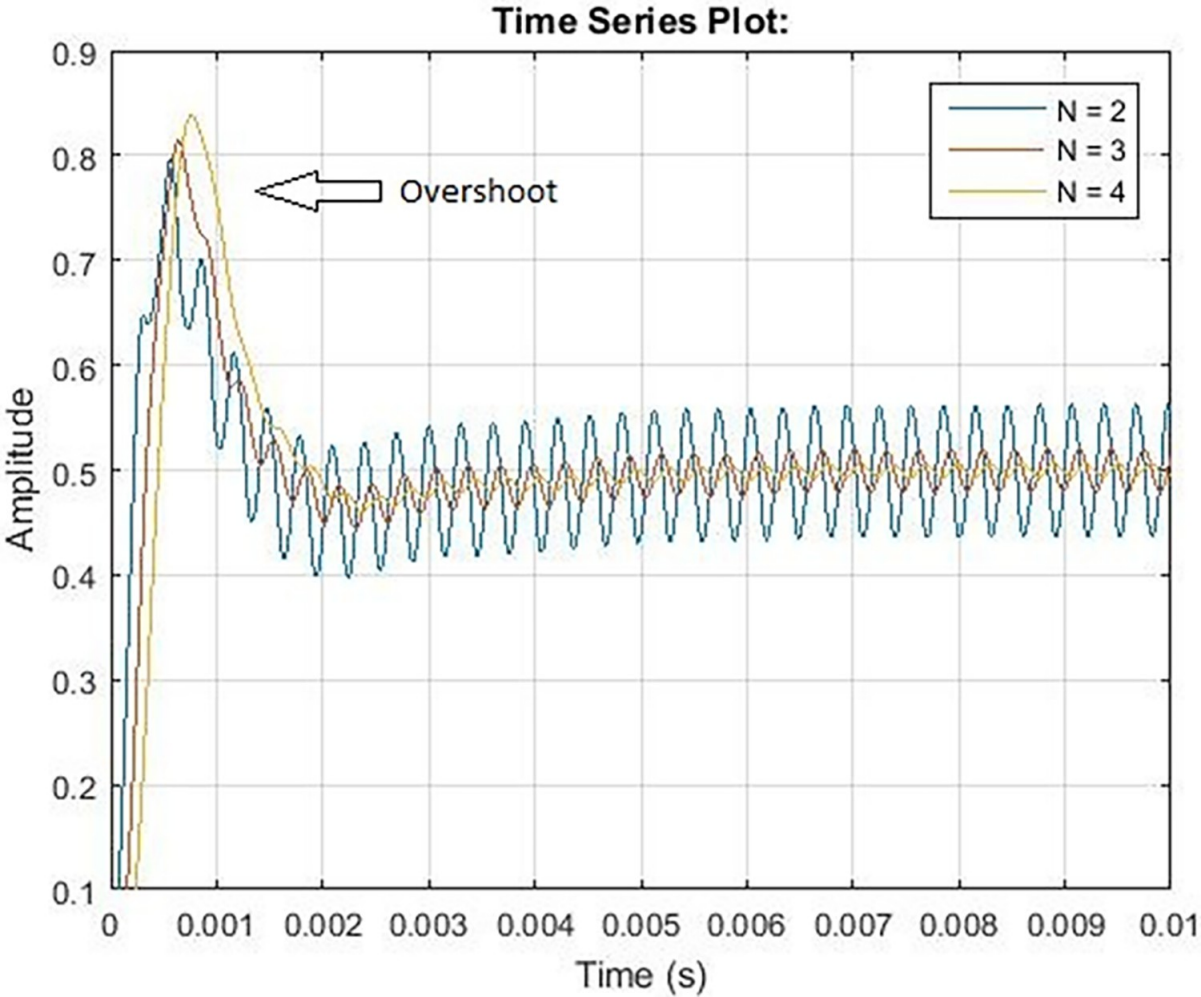
Overshoot and settling time for ‘N’.

**Fig 9:**
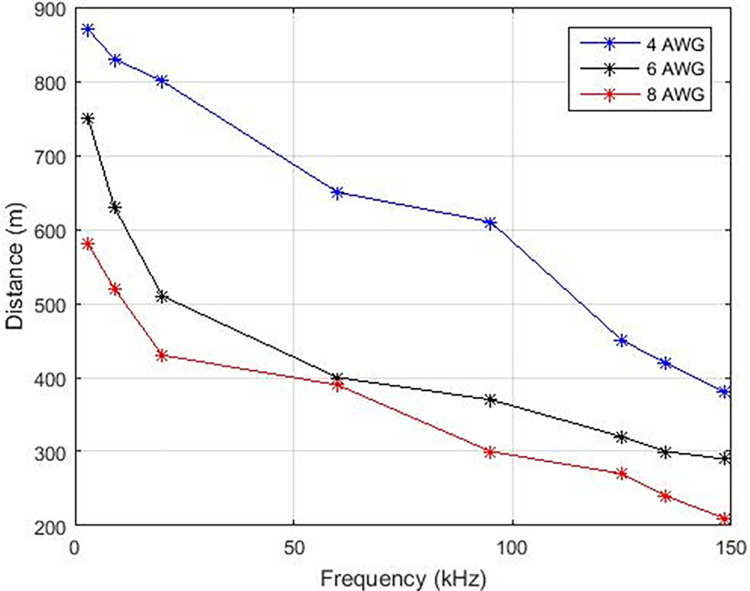
Distance v/s frequency (Copper).

**Fig 10:**
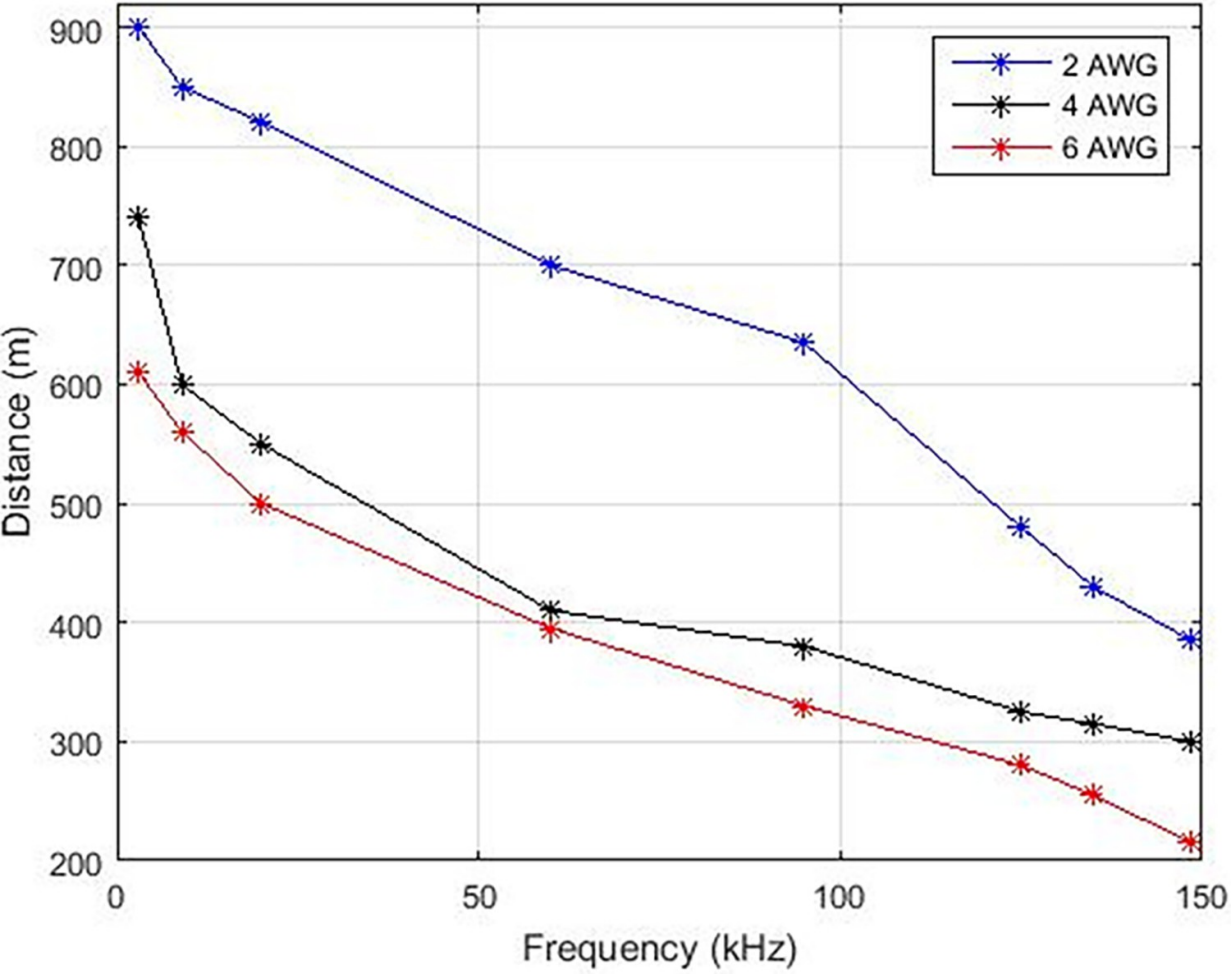
Distance v/s Frequency (ACSR).

In this manuscript, various carrier frequencies are examined to ascertain the transmission range over copper and aluminum cables, as depicted in Figs 8 and 9 correspondingly, which delineates that the transmission of higher carrier frequencies leads to reduced distance under the influence of a power signal. With the increment of frequency from 3 KHz, there is a consequent decrease in distance, resulting in a negative incline as portrayed in Figs 9 and 10. Augmenting the carrier frequency amplifies noise within the transmission medium, consequently impacting the stability and efficacy of data transmission. Conversely, enhancing the wire gauge, denoting the wire diameter, enables an extended distance for data transmission. Both copper and aluminum exhibit elongated line lengths when transitioning from 8 AWG to 4 AWG wire diameter.

The assessment of interference in the communication pathway between the sent and received signals was performed to establish the upper boundaries of transmission distances for various carrier frequencies, as illustrated in Table 6. In this table, the maximum permissible lengths of copper and aluminum lines are presented for different gauges that are deemed appropriate for Power Line Communication (PLC) applications. The transmission speeds attained through this particular PLC system surpass those reported in references [8,9,17,22].

However, diverse diameters of aluminum and copper wire indicate that copper wires of 4AWG, 6AWG, and 8AWG share equivalent lengths with aluminum wires of 2AWG, 4AWG, and 6AWG, respectively. The Power Line Carrier Communication Simulink model illustrates the tangible effects of the system on the household power line. A graphical representation displaying the bit error rate in relation to the data rate is generated across various levels of start band attenuation and stop band attenuation within a Butterworth filter.

**Fig 11:**
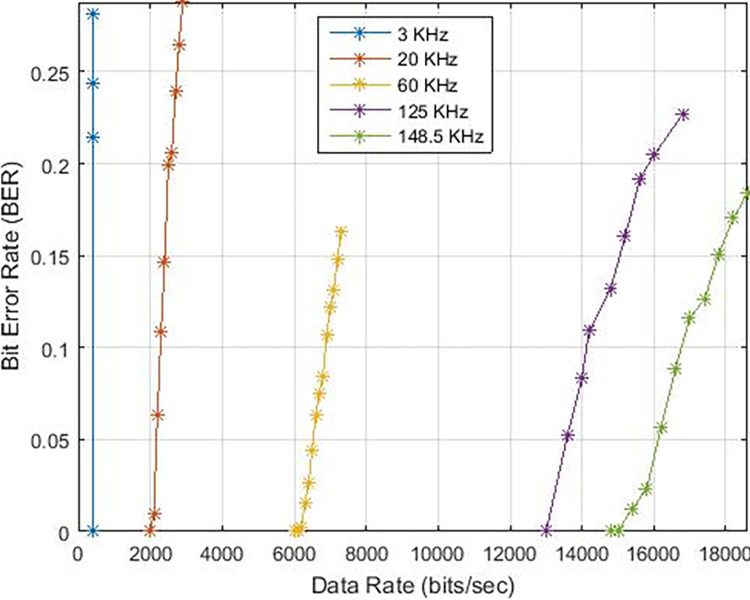
Data-Rate v/s BER.

**Fig 12:**
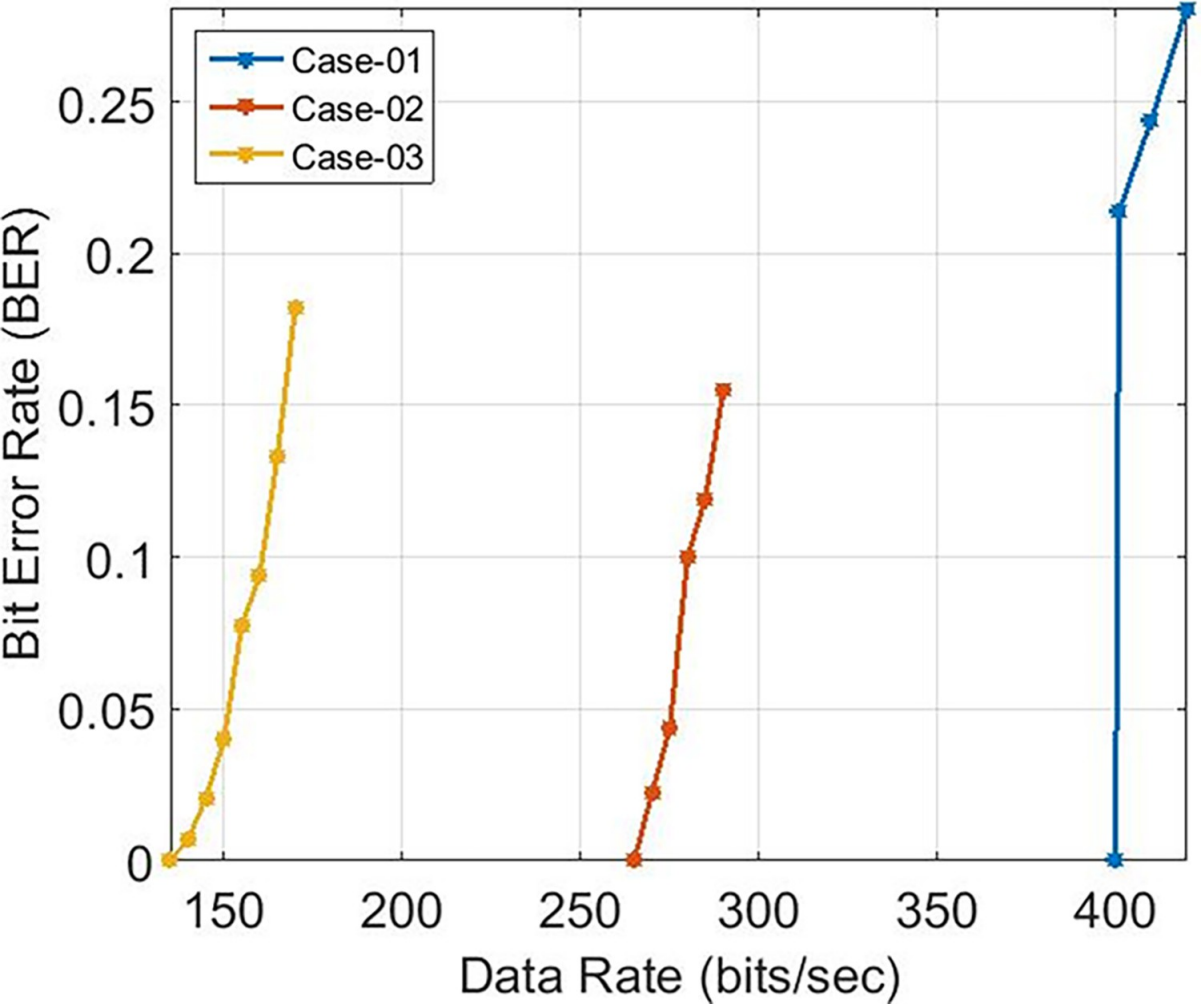
Data-Rate v/s BER for 3 KHz.

**Fig 13:**
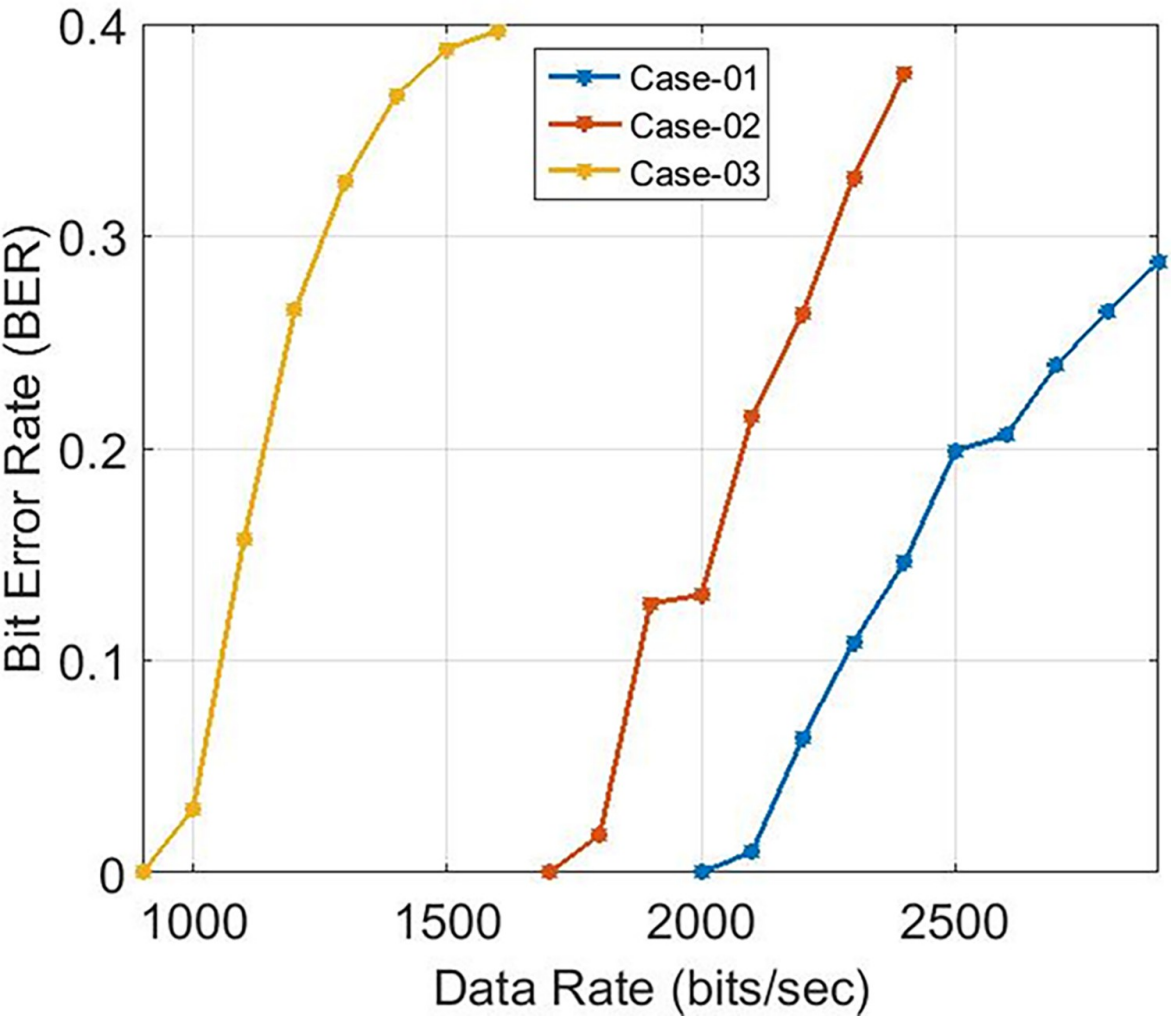
Data-Rate v/s BER for 20 KHz.

**Fig 14:**
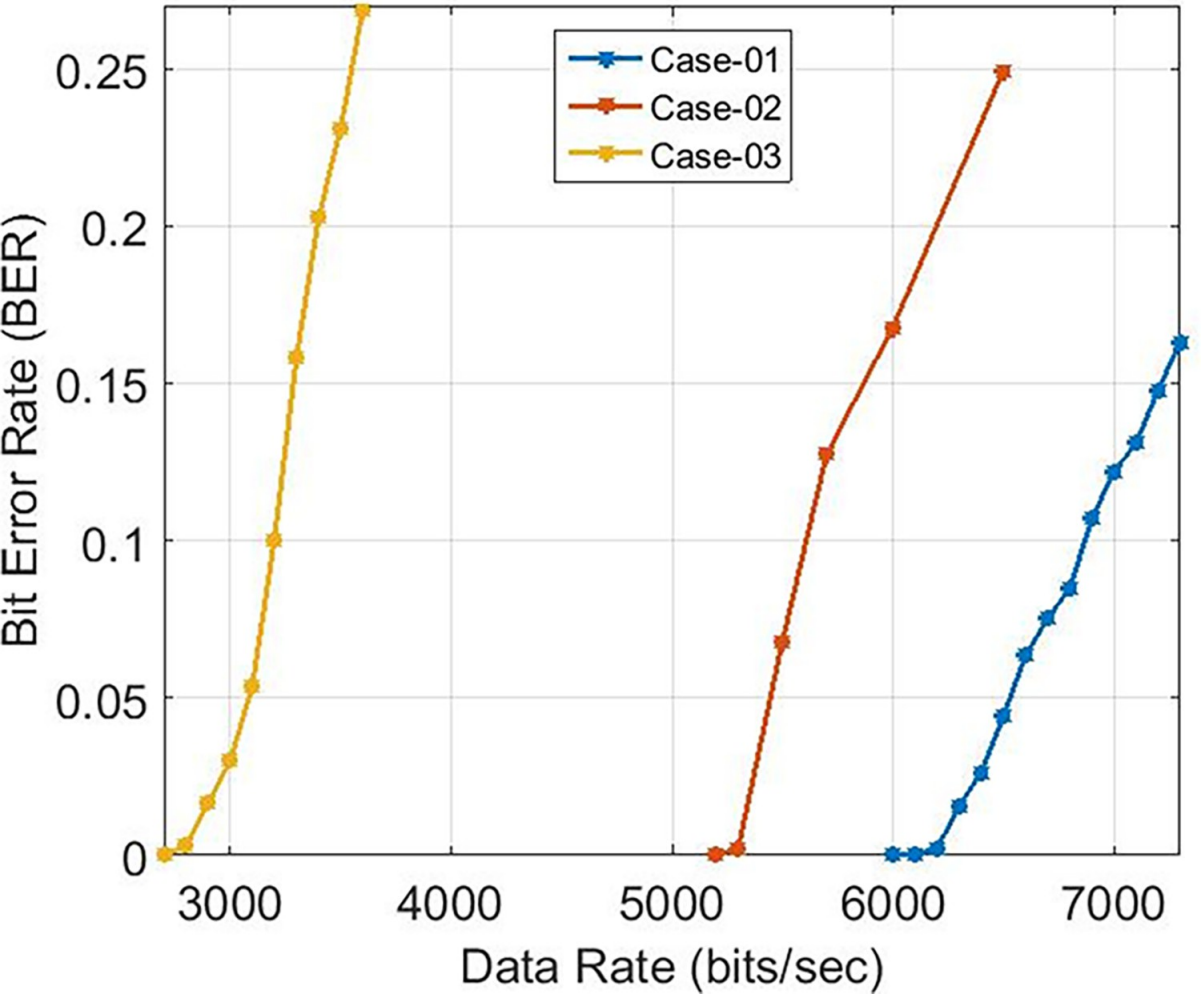
Data-Rate v/s BER for 60 KHz.

**Fig 15:**
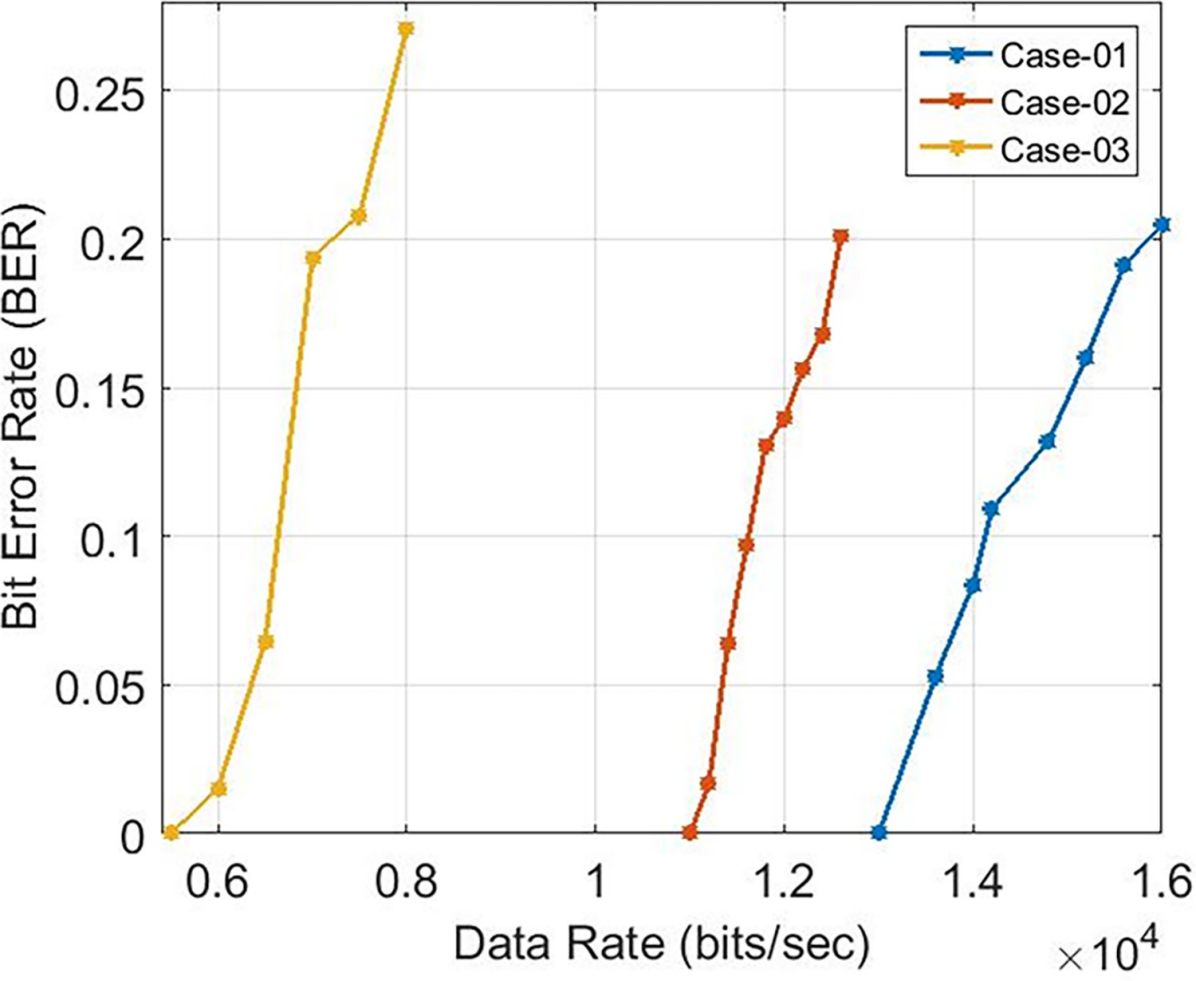
Data-Rate v/s BER for 125 KHz.

**Fig 16:**
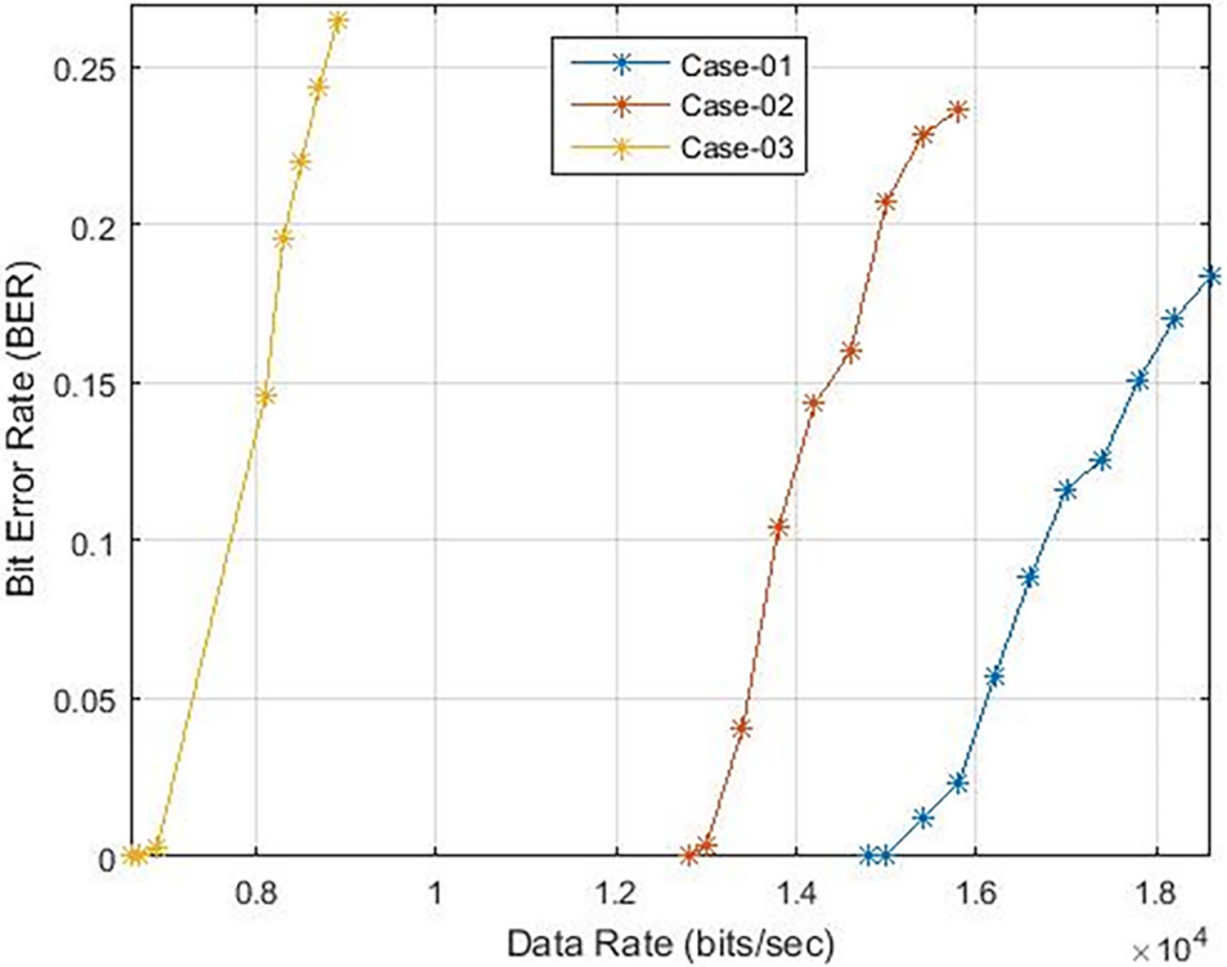
Data-Rate v/s BER for 148.5 KHz.

**Fig 17:**
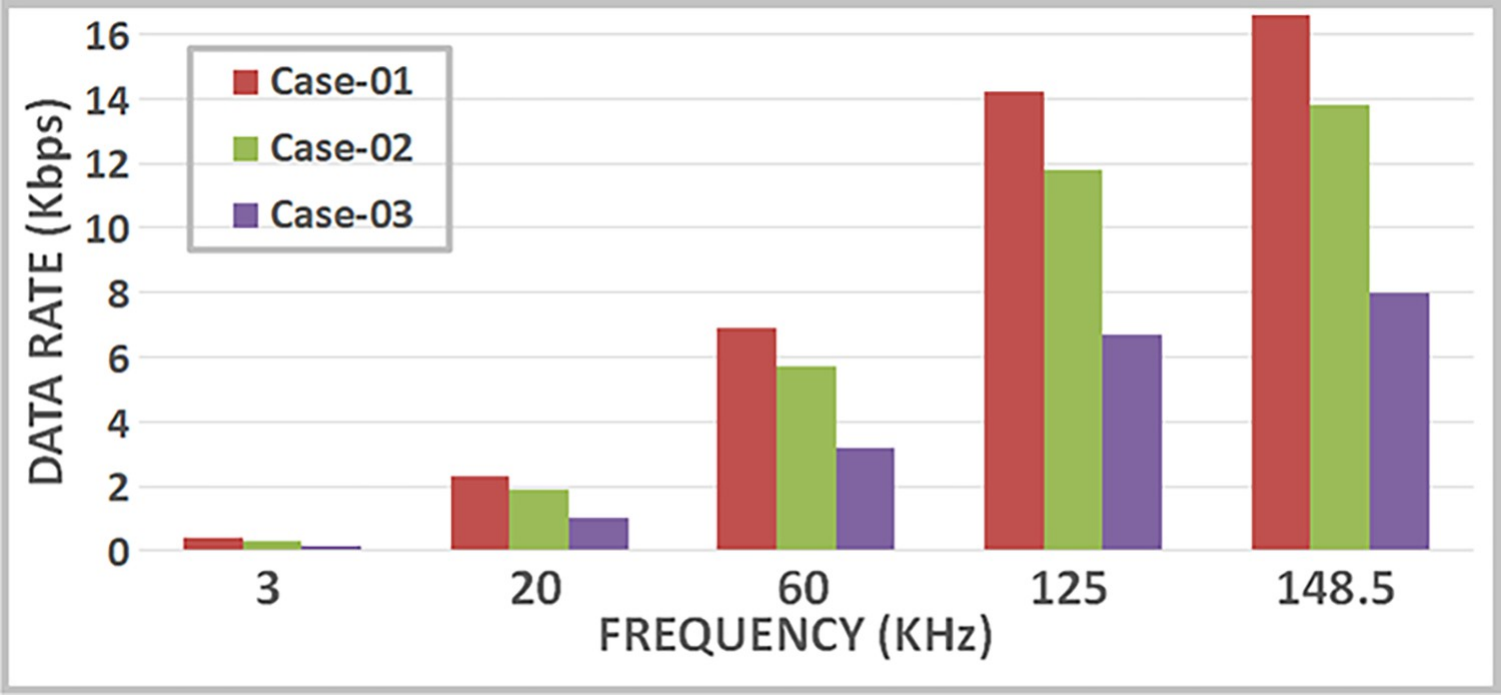
PLC data rates.

These findings indicate that the bit error rate is influenced by the demodulator rather than the characteristics of the cable such as length, diameter, and type. The data rates achievable for PLC under varying carrier frequencies are illustrated in Fig 11, with δ pass band = 37 and δ stop band = -40 for Case-01. The demodulator model for PLC is configured with three different pass band and stop band values to assess the effect of demodulator parameters on the reception. Consequently, δ pass band = 47 and δ stop band = -50 for Case-02, and δ pass band = 67 and δ stop band = -70 for Case-03. Fig 11 depicts the bit error rates obtained from our Simulink model at diverse data rates, showcasing the impact of different carrier frequencies and filter configurations. Particularly noteworthy is the observation that higher frequencies result in variations in bit error rates, especially for data rates exceeding 3 KHz, indicating that data rates below 0.4 Kbps are deemed acceptable, as evidenced in Fig 11. Sending bits beyond 0.4 Kbps can introduce errors at the reception end, leading to communication failure between the transmitter and receiver at a BER of 0.5. The proposed PLC model functions across various carrier frequencies (3 KHz, 20 KHz, 60 KHz, 125 KHz, or 148.5 KHz), providing corresponding data rates of 2.3 Kbps, 6.9 Kbps, 14.2 Kbps, or 16.6 Kbps respectively, as depicted in Fig 17, where higher carrier frequencies are associated with increased data rates. BER depicted in Fig 12 portrays the relationship between BER and data rate for a 3 KHz carrier frequency, demonstrating notable fluctuations in bit error rates. Extending this examination to carrier frequencies of 20 KHz, 60 KHz, 125 KHz, and 148.5 KHz are Fig 13–16, revealing that elevated data rates correlate with higher BER levels.

**Fig 18:**
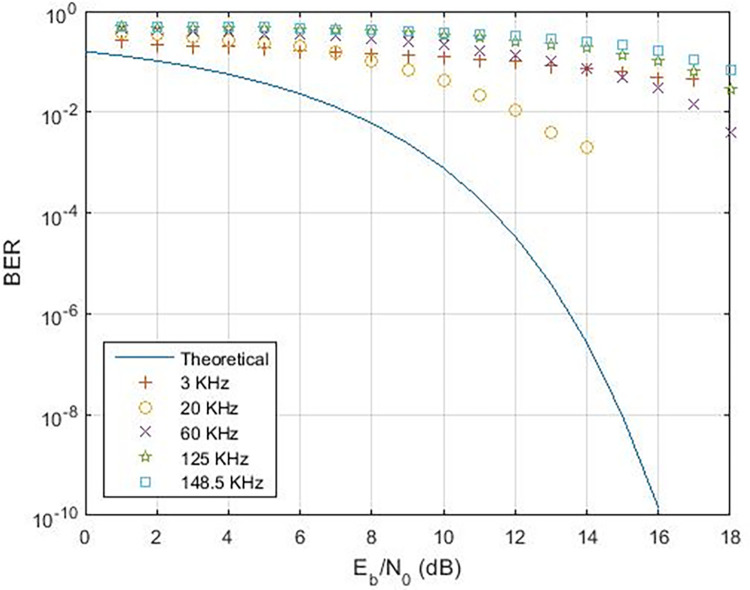
BER v/s SNR for Case-01.

**Fig 19:**
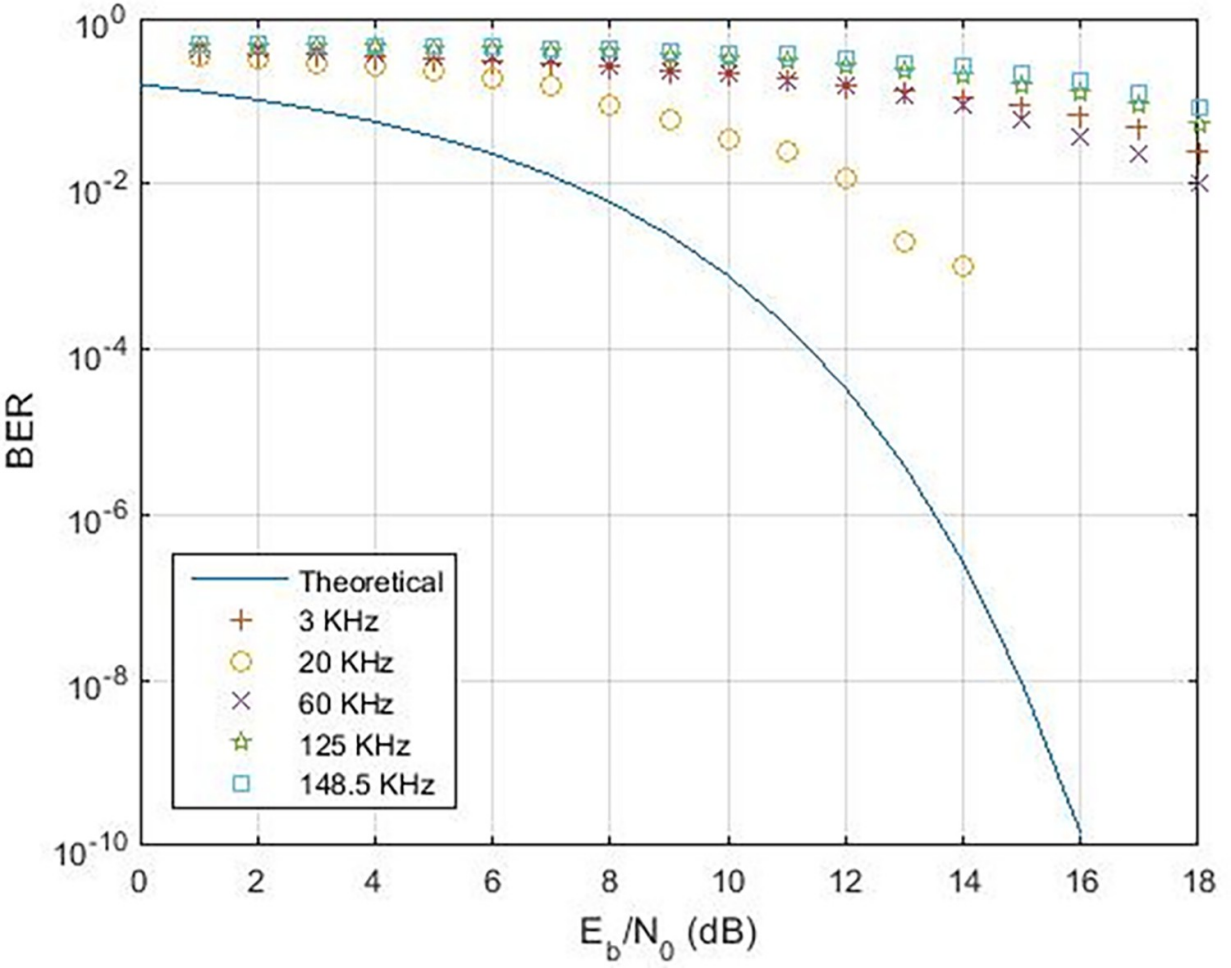
BER v/s SNR for Case-02.

**Fig 20:**
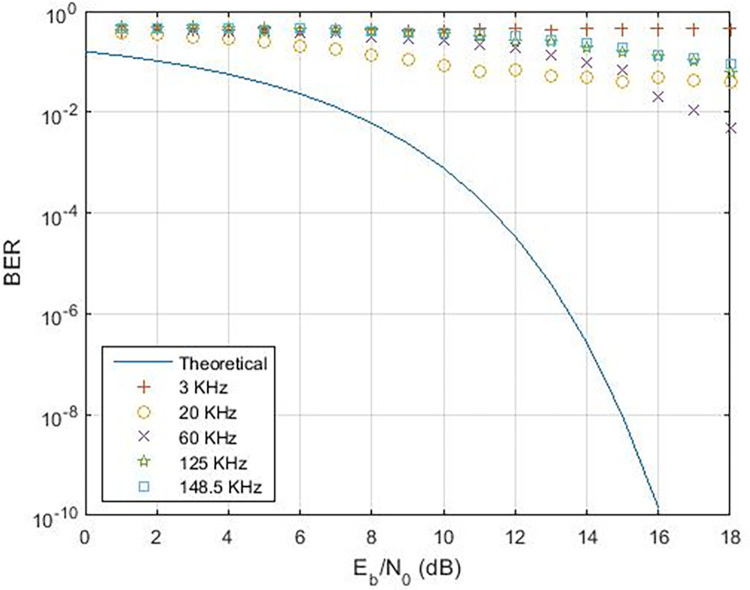
BER vs. SNR for Case-03.

When examining various instances, Fig 17 illustrates the range of data rates for case-01, spanning from 0.4 Kbps to 16.6 Kbps. The comparison with references [11,24] reveals that the proposed Power Line Communication (PLC) model attains superior data rates in contrast to the former but lags behind the latter, as outlined in Table 2. Fig 18 showcases the response of the proposed PLC model to noise at narrowband carrier frequencies, impacting the communication process. The presence of noise results in variations in Bit Error Rate (BER) as the frequency transitions from 3 KHz to 20 KHz. Notably, at 3 KHz, the impact of noise on errors is evident. As the frequency increases, the BER rises, subsequently declining gradually with improved Signal-to-Noise Ratio (SNR). However, higher frequencies exhibit a slower decline in BER due to the presence of Alternating Current (AC) signal noise. Upon scrutinizing case-02 and case-03 in terms of BER versus SNR (depicted in Figs 19 and 20), subtle variances become apparent, particularly at 20 KHz in Case-03.

**Fig 21:**
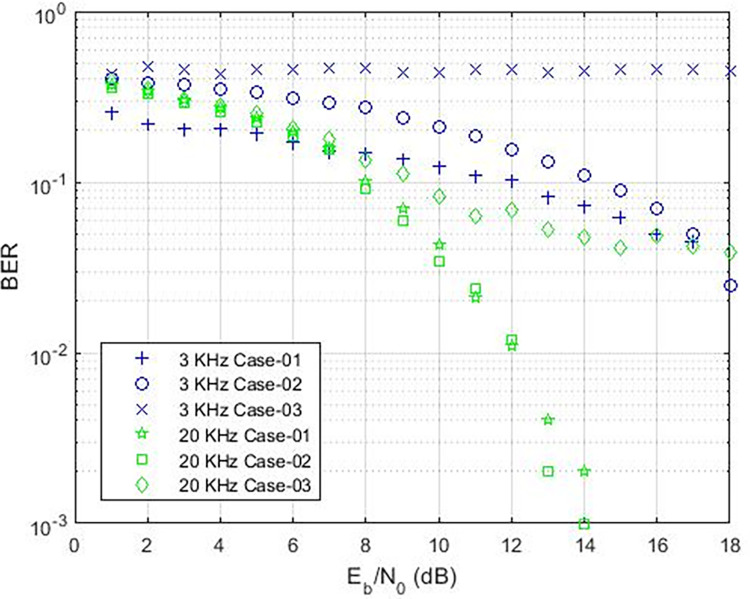
BER vs. SNR comparison between 3 KHz and 20 KHz.

**Fig 22:**
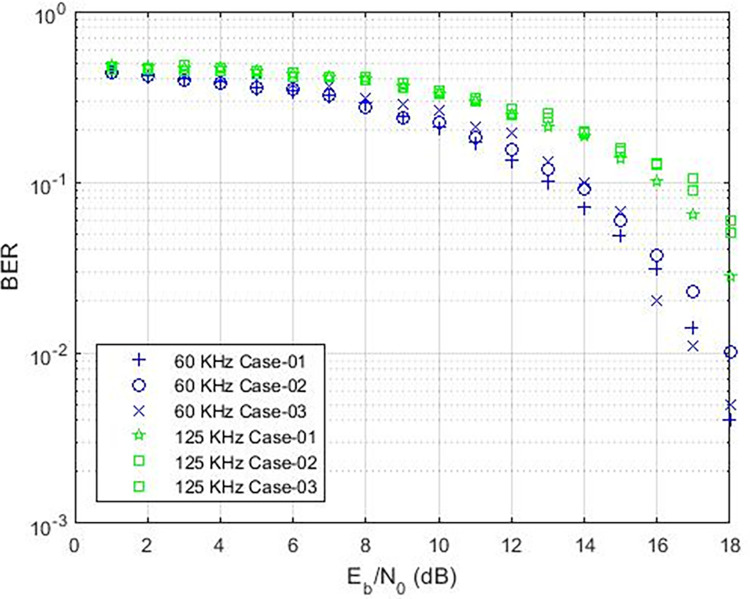
BER vs. SNR comparison between 60 KHz and 125 KHz.

**Fig 23:**
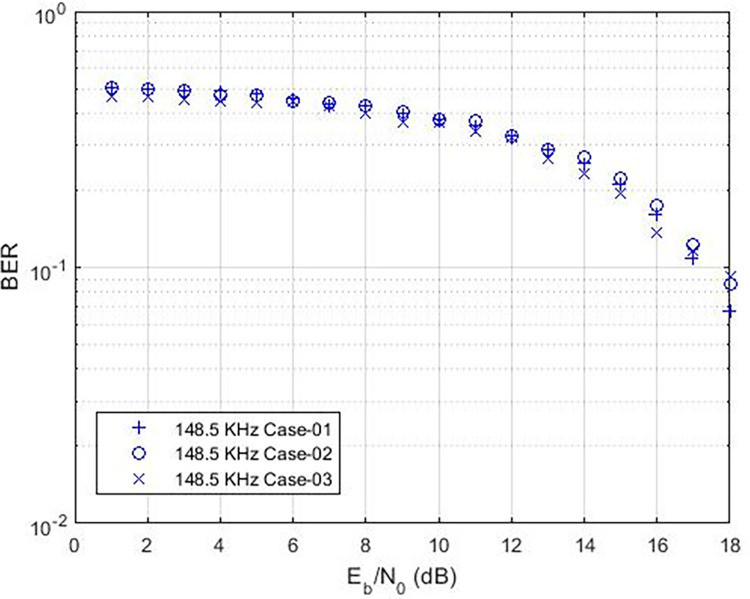
BER vs. SNR for 148 KHz.

The utilization of higher pass band and stop band values in the demodulator may not produce optimal outcomes, as indicated. Simulations validate the robustness of FSK PLC against signal attenuation and interference at low voltage levels. The carrier frequency of 20 KHz closely aligns with the expected values in the BER v/s SNR response. A comparison of BER v/s SNR for 3 KHz and 20 KHz is illustrated in Fig 21. The stability at 20 KHz surpasses that at 3 KHz. Nevertheless, modifications to the pass band and stop band introduce instability at 20 KHz (case-02), while 3 KHz remains steady. The selection of appropriate demodulation parameters is imperative for ensuring dependable communication within home area networks. PLC exhibits greater stability at 60 KHz compared to 125 KHz, as depicted in Fig 22. Higher carrier frequencies necessitate a higher SNR due to low voltage levels, a characteristic essential for reliable communication. Furthermore, marginal shifts in BER are observed at 148.5 KHz, as shown in Fig 23.

## V. Discussion

Power Line Communication (PLC) represents an innovative technological advancement employed in Home Area Networks (HANs), smart grids, the Internet of Things (IoT), and various other applications. The technology presents a myriad of benefits, such as a streamlined installation process and reduced costs. Nevertheless, prior to implementation within a household, specific challenges concerning PLC Communication must be effectively tackled. One of the predominant obstacles encountered in PLC communication pertains to its vulnerability to interference and disturbances within the power line originating from other electrical devices, consequently resulting in diminished data rates. Consequently, the efficacy of a PLC system hinges on the caliber of the data transmission medium. In conclusion, it is imperative to conduct trials of PLC communication utilizing the existing wiring infrastructure, complemented by a simulator or model to pinpoint potential issues and outcomes within authentic Home Area Network (HAN) settings. While PLCC Simulink represents a relatively recent innovation, there exist numerous areas warranting further exploration in future research endeavors on PLC communication, aimed at enhancing its performance and reliability through the exploration of diverse modulation techniques and the incorporation of additional filters to ameliorate bit error rates.

## VI. Conclusion

In this manuscript, a Simulink PLC model is utilized to assess the efficacy of power line carrier communication within a residential area network. A pragmatic transmission line model along with additive white Gaussian noise is incorporated to facilitate real-time scrutiny. The optimal modulation scheme for PLC technology is Frequency Shift Keying (FSK) due to its robustness and stability. It has been observed that FSK modulation is minimally impacted by noise within the transmission medium. The influence of higher frequencies was evaluated through communication across distinct carrier frequencies. Elevated carrier frequencies exhibit increased noise within the transmission medium, consequently leading to diminished distance coverage. Specifically, carrier frequencies ranging from 3 KHz to 148.5 KHz resulted in a reduction of line length from 870 to 380 meters for a 4 American Wire Gauge (AWG) wire. Likewise, diminishing the cable diameter also correlates with reduced cable distance. To achieve a cable length akin to 4 AWG copper cable, 6 AWG and 8 AWG aluminum cables are necessitated. Correspondingly, carrier frequency influenced the data rates for PLC technology in the narrow band. Augmented carrier frequencies yielded enhanced data rates within a residential area network. Data rates associated with a carrier frequency of 148.5 KHz surpass those of 3 KHz. Furthermore, an examination of three demodulation scenarios was conducted to elucidate the impact of altering pass band and stop band attenuation. Elevated pass band and stop band attenuation levels yielded lowered data rates accompanied by an unstable Bit Error Rate (BER) versus Signal-to-Noise Ratio (SNR). **Furthermore, escalating the filter’s order resulted in prolonged settling time and overshoots. Ultimately, it is deduced that FSK modulation with a carrier frequency of 20 KHz is the most suitable technique within a distance range of 700 to 390 meters, offering data rates of 2.3 Kilobits per second (Kbps) for aluminum or copper cables with a pass band deviation (δ) of 37 and stop band deviation (δ) of -40 (Case-01) within a residential area network.**

## References

[1] Aderibole A. O., Saathoff E. K., Kircher K. J., Leeb S. B., Norford L. K., Power line communication for low-bandwidth control and sensing, IEEE Transactions on Power Delivery 37 (3) (2022) 2172–2181. doi: 10.1109/TPWRD.2021.3106585

[2] Sendin A., Arzuaga T., Urrutia I., Berganza I., Fernandez A., Marron L., Llano A., Arzuaga A., Adaptation of powerline communications-based smart metering deployments to the requirements of smart grids, Energies 8 (12) (2015) 13481–13507.

[3] Ali S.U., Waqar A., Aamir M., Qaisar S.M. and Iqbal J., 2023. Model predictive control of consensus-based energy management system for DC microgrid. *PloS one*, 18(1), p.e0278110. 36662901 10.1371/journal.pone.0278110 PMC9858890

[4] Iqbal J. and Khan Z.H., 2017. The potential role of renewable energy sources in robot’s power system: A case study of Pakistan. *Renewable and Sustainable Energy Reviews*, 75, pp.106–122.

[5] Hassan M.U., Nawaz M.I. and Iqbal J., 2017, November. Towards autonomous cleaning of photovoltaic modules: Design and realization of a robotic cleaner. In *2017 First International Conference on Latest trends in Electrical Engineering and Computing Technologies (INTELLECT)* (pp. 1–6). IEEE.

[6] Ashraf M., Gulraiz A., Zaidi S.S.H., Ashraf F. and Khan B.M., 2022, November. Wind’s data analysis for its accurate prediction in smart grid systems. In *2022 Third International Conference on Latest trends in Electrical Engineering and Computing Technologies (INTELLECT)* (pp. 1–5). IEEE.

[7] Ashraf M., Raza B., Arshad M., Khan B.M. and Zaidi S.S.H., 2024. Performance enhancement of short-term wind speed forecasting model using Realtime data. *Plos one*, 19(5), p.e0302664. doi: 10.1371/journal.pone.0302664 38820359 PMC11142572

[8] Osman W. R. S., Nisar K., Altrad A. M., Evaluation of broadband plc technology over malaysia’s indoor power line network, in: 2014 2nd international conference on electronic design (ICED), IEEE, 2014, pp. 275–280.

[9] Cataliotti A., Cosentino V., Di Cara D., Tine G., Oil-filled mv/lv powertransformer behavior in narrow-band power-line communication systems, IEEE Transactions on Instrumentation and Measurement 61 (10) (2012) 2642–2652.

[10] Buayairaksa S., Thepphaeng S., Pirak C., On the performance of g3 power line communication network with smart energy meter, in: 2013 10th International Conference on Electrical Engineering/Electronics, Computer, Telecommunications and Information Technology, IEEE, 2013, pp. 1–5.

[11] Aalamifar F., Lampe L., Optimized data acquisition point placement for an advanced metering infrastructure based on power line communication technology, IEEE Access 6 (2018) 45347–45358.

[12] Llano A., De La Vega D., Angulo I., Marron L., Impact of channel disturbances on current narrowband power line communications and lessons to be learnt for the future technologies, IEEE Access 7 (2019) 83797–83811.

[13] Sharma D., Dubey A., Mishra S., Mallik R. K., A frequency control strategy using power line communication in a smart microgrid, IEEE Access 7 (2019) 21712–21721.

[14] Tonello A. M., Versolatto F., Pittolo A., In-home power line communication channel: Statistical characterization, IEEE Transactions on Communications 62 (6) (2014) 2096–2106.

[15] Kharraz M. A. O., Picard D., Serhir M., Lavenu C., Jensen P., Measurement methods of outdoor low-voltage cable characteristics for narrowband power line communication, IEEE Transactions on Power Delivery 34 (5) (2018) 1818–1826.

[16] Castor L. R., Natale R., Silva J. A., Segatto M. E., Experimental investigation of broadband power line communication modems for onshore oil & gas industry: A preliminary analysis, in: 18th IEEE International Symposium on Power Line Communications and Its Applications, IEEE, 2014, pp. 244–248.

[17] Pinero P., Cortes J. A., Malgosa J., Cañete F. J., Manzanares P., Díez L., Analysis and improvement of multicast communications in homeplug av-based in-home networks, Computer Networks 62 (2014) 89–100.

[18] Merkulov A. G., Shuvalov V. P., The perspectives and practice of plc homeplug av modems application in the network devices and industrial tools, in: 2019 1st Global Power, Energy and Communication Conference (GPECOM), IEEE, 2019, pp. 46–49.

[19] Orgon M., Stefanicka M., Schmidt I., Zolotova I., Cupkova D., Testing home plc network in multi-storey house, in: 2019 11th International Congress on Ultra-Modern Telecommunications and Control Systems and Workshops (ICUMT), IEEE, 2019, pp. 1–6.

[20] Hallak G., Berners M., Mengi A., Planning approach towards optimal performance and cost of g. hn broadband plc access networks, in: 2020 IEEE International Symposium on Power Line Communications and Its Applications (ISPLC), IEEE, 2020, pp. 1–6.

[21] Mlynek P., Fujdiak R., Misurec J., Slacik J., Experimental measurements of noise influence on narrowband power line communication, in: 2016 8th International Congress on Ultra-Modern Telecommunications and Control Systems and Workshops (ICUMT), IEEE, 2016, pp. 94–100.

[22] Uribe-Perez N., Angulo I., de la Vega D., Arzuaga T., Arrinda A., Fernandez I., On-field evaluation of the performance of ip-based data transmission over narrowband plc for smart grid applications, International Journal of Electrical Power & Energy Systems 100 (2018) 350–364.

[23] Chen S., Yang Z., A low cost single phase plc watt-hour meter based on soc, in: 2012 2nd International Conference on Consumer Electronics, Communications and Networks (CECNet), IEEE, 2012, pp. 1523–1526.

[24] Hsieh S.-C., Ku T.-T., Tsai J.-C., Lin C.-H., Chen C.-S., broadcasting control of intelligent air conditioners using power line carrier technology, in: 2014 IEEE/IAS 50th Industrial & Commercial Power Systems Technical Conference, IEEE, 2014, pp. 1–6.

[25] Mlynek P., Misurec J., Kolka Z., Slacik J., Fujdiak R., Narrowband power line communication for smart metering and street lighting control, IFAC-PapersOnLine 48 (4) (2015) 215–219.

[26] Gozalpour F., Yavari M., An improved fsk-modulated class-e power and data transmitter for biomedical implants, AEU-International Journal of Electronics and Communications (2023) 154786.

[27] Peck M., Alvarez G., Coleman B., Moradi H., Forest M., Aalo V., Modeling and analysis of power line communications for application in smart grid, arXiv preprint arXiv: 1709.06883 (2017).

[28] Y. Zhou, Y. Wang, Research on modulation technology of low-voltage power line carrier communication, in: 2013 Fourth Global Congress on Intelligent Systems, IEEE, 2013, pp. 296–298.

[29] Xie X., Zuo H., Wei S., Wu Q., Motion-induced noise detection of electrode-pair towed antennas using helix coil sensors, Electronics 12 (7) (2023) 1677.

[30] Fan L.-F., Wang Z.-Y., Huang L., Wang Y.-H., Measurement of twolayer medium dielectric property using a novel parameters model in radio frequency, International Journal of Circuit Theory and Applications (2023).

[31] Glover J. D., Sarma M. S., Overbye T., Power system analysis & design, SI version, Cengage Learning, 2012.

[32] Kaufmann F., Strugulea M., Höltgen C., Roth S. and Schmidt M., 2023. Seam properties of overlap welding strategies from copper to aluminum using green laser radiation for battery tab connections in electric vehicles. *Materials*, 16(3), p.1069. doi: 10.3390/ma16031069 36770076 PMC9921288

